# Time of day-dependent alterations of ferroptosis in LPS-induced myocardial injury via Bmal-1/AKT/ Nrf2 in rat and H9c2 cell

**DOI:** 10.1016/j.heliyon.2024.e37088

**Published:** 2024-08-31

**Authors:** Pei Ying-hao, Yang Yu-shan, Cheng Song-yi, Jiang Hua, Yu Peng, Chen Xiao-hu

**Affiliations:** a Department of Intensive Care Unit, Jiangsu Province Hospital of Chinese Medicine, Affiliated Hospital of Nanjing University of Chinese Medicine, Jiangsu Province, Nanjing, China; b Department of Cardiology, Jiangsu Province Hospital of Chinese Medicine, Affiliated Hospital of Nanjing University of Chinese Medicine, Jiangsu Province, Nanjing, China; c Department of Cardiology, the People's Hospital of Qingyang City, Gansu Province, China; d Department of Cardiology, Nanjing Hospital of Chinese Medicine affiliated to Nanjing university of Chinese medicine, Jiangsu Province, Nanjing, China

**Keywords:** Ferroptosis, LPS-Induced myocardial injury, Circadian, Endotoxemia, Bmal-1/AKT/Nrf2 pathway

## Abstract

**Background:**

One of the most prevalent causes of death in sepsis is sepsis-induced cardiomyopathy (SICM). Circadian disruption is involved in the progress of sepsis. However, the molecular mechanism remains unclear.

**Methods:**

Here, we built LPS-induced SICM *in-vivo* and *in-vitro* models. LPS was administrated at the particular Zeitgeber times (ZT), ZT4-ZT10-ZT16-ZT22 and ZT10-ZT22 in *vivo* and *vitro* experiments, respectively.

**Results:**

In *vivo* experiment, injection of LPS at ZT10 induced higher infiltration of inflammatory cells and content of intracellular Fe^2+^, and lower level of Glutathione peroxidase 4 (GPX4) and cardiac function than other ZTs (P < 0.05), which indicated that myocardial ferroptosis in septic rat presented a time of day-dependent manner. Bmal-1 protein and mRNA levels of injection of LPS at ZT10 were lower than those at other three ZTs (P < 0.05). The ratios of pAKT/AKT at ZT4 and ZT10 LPS injection were lower than those at ZT16 and ZT22 (P < 0.05). Nrf2 protein levels at ZT10 LPS injection were lower than those at other three ZTs (P < 0.05). These results indicated that the circadian of Bmal-1 and its downstream AKT/Nrf2 pathway in rat heart were inhibited under SICM condition. Consistent with *in-vivo* experiment, we found LPS could significantly reduce the expressions of Bmal-1 protein and mRNA in H9c2 cell. Up-regulation of Bmal-1 could reduce the cell death, oxidative stress, ferroptosis and activation of AKT/Nrf2 pathway at both ZT10 and ZT22 LPS administration. Conversely, its down-regulation presented opposite effects. AKT siRNA could weaken the effect of Bmal-1 pcDNA.

**Conclusion:**

Ferroptosis presented the time of day-dependent manners via Bmal-1/AKT/Nrf2 in *vivo* and *vitro* models of SICM.

## 1 Introduction

Sepsis represents a significant global cause of morbidity and mortality. Due to recent epidemiological study, sepsis causes 11 million deaths each year [1]. Sepsis can progress to multiple organ dysfunction and heart is one of the most frequently disturbed organs [2]. Sepsis-induced cardiomyopathy (SICM) represents a significant contributor to mortality in septic patients [3]. The prevalence of SICM in septic patients ranges from 10 to 70 % [4]. SICM is precipitated by a constellation of factors, including stunning of myocardium, myocardial ischemia, mitochondrial dysfunction, coronary microvascular dysfunction, dysregulation of the adrenergic system, and inflammation damage [5].

Sepsis is considered to result from an imbalance in the immune response [6]. Immune cells demonstrate properties that vary depending on the time of day and are regulated via the circadian genes, including *CLOCK* and *BMAL-1* [7]. These clock genes build feedback loops of autoregulatory transcription-translation to generate molecular circadian rhythms [8]. In macrophages, the regulation of immune checkpoint pathways is mediated by various circadian genes. During experimental sepsis, Bmal1, the core clock gene, regulates host immune responses [9]. A previous study showed that the non-septic patients maintained circadian rhythm of clock genes, while in septic cases it was blunted [10]. In septic shock patients, molecular circadian rhythms of immune cells were observed to be markedly decreased and altered [7]. In a rat model of endotoxemia, which simulates septic conditions, lipopolysaccharide (LPS) has been shown to disrupt the systems of circadian rhythm and presented effects on cardiac clock genes [11]. These studies indicate that circadian disruption is related to the onset and progress of sepsis. However, the mechanism remains unclear.

Ferroptosis is a new kind of programmed cell death characterized by insufficient cellular scavenging capacity and Fe^2+^-dependent lipid peroxidation [12]. Oxidative stress is posited as a primary instigator of ferroptosis. The overproduction of intracellular iron induces excessive reactive oxygen species (ROS), which subsequently triggers cellular injury and death [13]. It was reported that LPS administration elevated the ferroptotic indexes in the H9c2 cardiomyocyte of *in-vitro* endotoxemia models and the rat heart of *in-vivo* models [14]. Bmal-1, a fundamental part of the circadian clock, serves as a crucial modulator of cellular reactivity associated with cardiovascular function. Research in cardiomyocytes indicates that Bmal-1 exerts effects against the production of ROS [15]. Based on these findings, we hypothesized that LPS-induced ferroptosis presented as the time of day-dependent alterations mediated by the circadian disruption of Bmal-1 and its downstream pathway.

Herein, our study aimed to determine whether ferroptosis exhibits diurnal variation in the context of sepsis and to elucidate the underlying mechanisms linking ferroptosis and circadian disruption. This investigation was conducted using both in *vivo* and in *vitro* models of LPS-induced SICM.

## 2 Materials and methods

### 2.1 In vivo rat model of SICM

Adult male Sprague-Dawley rats, weighing between 220 and 250 g and aged 8 weeks, were procured from Beijing Huafukang Biological Technology Co., Ltd. The rats were housed in groups of eight under controlled environmental conditions, with a temperature maintained at 22 ± 2 °C and humidity levels between 60 % and 70 %. A 12-h light-dark cycle was implemented. The water and food were provided ad libitum. All procedures adhered to the guidelines established by the National Research Council's Guide for the Care and Use of Laboratory Animals.

All rats were randomly assigned to either the control group or the SICM group, with each group comprising eight rats. The rat model of SICM was built by 10 mg/kg LPS intraperitoneal injection. The rats of the control group received an equivalent volume of normal saline via the intraperitoneal injection. Dose of LPS was based on our previous experiments and other studies [16]. Zeitgeber time (ZT) is a temporal unit derived from the periodicity of a zeitgeber. In our study, the lights-on and lights-off were defined as ZT0 and ZT12, respectively. For evaluating the time-dependent effects of LPS, rats were intraperitoneal injected with LPS at ZT4, ZT10, ZT16 or ZT22 and sacrificed 2h after each injection. A total of 40 rats, 10 rats per ZT group, were used for 72 h survival rate analysis.

### 2.2 In vitro cell model of SICM

H9c2 cells, the rat cardiomyocyte cell line, were obtained from the Cell Bank/Stem Cell bank of Chinese Sciences Academy in Shanghai. The cells were cultured in Dulbecco's modified Eagle medium supplemented with 1 % penicillin/streptomycin solution (Gibco-BRL, Grand Island, NY) and 10 % fetal bovine serum (Sigma, St Louis, MO). To achieve synchronization of the circadian clock, cells were exposed to 100 nM dexamethasone for a duration of 1 h as previously documented in the literature [17]. ZT0 was designated as 1 h post-administration of dexamethasone. Following synchronization of the circadian clock, 1 μg/mL LPS was added into the culture medium for a duration of 2 h to establish the in *vitro* SICM model at ZT10 and ZT22, respectively. Dose of LPS was based on our previous experiments and other studies [18]. Each sample represented an independent replicate, and each experiment was repeated at least 3 times.

### 2.3 Hematoxylin&eosin (H&E) staining

H&E was conducted in accordance with the methodology outlined in our previous study [19]. The transverse slice of the middle segment of a heart was stained with H&E for assessing myofilament morphology and infiltration of inflammatory cells.

### 2.4 Vital signs evaluation and echocardiography

A programmable sphygmomanometer (BP-2010E, Japan) was used to assess the mean arterial pressure (MAP) and heart rate (HR) via the tail-cuff method [20]. The temperature was measured via a probe inserted into the rat's rectum.

The details of transthoracic echocardiography was described in our previous study [19,21]. Briefly, echocardiography was carried out using a GE ultrasound system with a 12S linear array ultrasound transducer (USA, GE Vivid E95). M-mode echocardiography was employed to assess the cardiac dimensions and function.

### 2.5 Detection of iron in H9c2 cell and rat heart

The Iron Assay Kit (DojinDo, Japan) was utilized to quantify the Fe^2^⁺ concentration in rat heart tissue, following the protocol provided by the manufacturer. Briefly, a 100 mg sample of rat heart tissue was washed and homogenized in 1.3 mL of Assay Buffer, and centrifuged at 16,000g for 10 min at 4 °C using Foring technology. The concentration of Fe^2^⁺ in the resulting supernatants was then quantified using a microplate reader.

FerroOrange (DojinDo, Japan) was used to detect Fe^2+^ level. After LPS administration, cells were washed and treated with 1 mM FerroOrange for 30 min at 37 °C. Fluorescence images were obtained using Ts2-inverted fluorescent microscope (Nikon, Japan).

### 2.6 Cell transfection

Following the instructions of our previous study [22], oligonucleotides were transfected into cells. Briefly, we seeded H9c2 cells 24 h before transection. Then, the oligonucleotides (200 nM Bmal-1 siRNA and AKT siRNA, General Biol, China) and Lipofectamine 3000 transfection reagent (Invitrogen, USA) were diluted using Opti-MEM medium without antibiotics and serum for 48 h incubation.

### 2.7 Cell viability assay

Cell viability was assessed utilizing Trypan Blue staining following the protocol (Beyotime, Shanghai, China).

### 2.8 Measurements of MDA

Malondialdehyde (MDA) serves as a biomarker for lipid peroxidation. The MDA levels in H9c2 cells were quantified utilizing commercially available assay kits, following the established protocols from prior studies [19].

### 2.9 ROS measurement

Dihydroethidium (DHE) staining (Beyotime, Shanghai, China) was employed for the detection of ROS. The levels of ROS were assessed using fluorescence microscope (Ts2, Nikon, Japan) and flow cytometry (CytoFLEX, Beckman, United States).

### 2.10 Lipid peroxidation assessment

Liperfluo (DojinDo, Japan) was employed to quantify the contents of lipid peroxidation levels following the protocol. In brief, H9c2 cells were seeded, washed and pre-stained with 10uM Liperfluo for 30 min. After centrifugation, the supernatants were removed, and the cells were washed by serum‐free medium. The lipid peroxidation was analyzed by fluorescence microscope (Ts2, Nikon, Japan) and flow cytometry (CytoFLEX, Beckman, United States).

### 2.11 Western blotting analysis

Western blotting analysis was conducted in accordance with the methodology outlined in our previous study [22,23]. After determining the concentration of protein, the subcellular fractions and cell lysates were separated and subsequently transferred. Next, the membranes were blocked and incubated with anti-GPX4 (1:1000, ABCAM, ab252833), anti-Bma1 (1:1000, ABCAM, ab235577), anti-Nrf2 (1:2000, proteintech, 16396-1-AP), anti-p-AKT (Thr308) (1:1000, CST, 13038T), and anti-GAPDH (1:10000, proteintech, 60004-1-Ig), respectively. The bound antibodies were tested with goat-anti-rabbit IgG-HRP (1:5000, Kaiji Biotech., KGAA35). The chemiluminescence ECL kit (Applygen Technologies) was used to develop the bands. The ImageJ system was used to evaluate the relative expressions of each protein normalized to GAPDH.

### 2.12 qRT-PCR analysis

Total RNAs were extracted using TRIzol reagent. RT-qPCR was subsequently conducted in accordance with the protocol in our prior researches [22,23]. The relative expression levels of RNAs were determined using the ΔΔCT method. The primer sequences are as follows: AKT: sense: 5-GCCTCTGCTTTGTCATGGAG-3, Antisense: 5-AGCATGAGGTTCTCCAGCTT-3; Bmal-1: sense: 5-CGGCGCTCTTTCTTCTGTAG-3, Antisense: 5-GTAGCCTGTGCTGTGGATTG-3; GAPDH: sense: 5-AAGATGGTGAAGGTCGGTGT-3, Antisense: 5-GCTTCCCATTCTCAGCCTTG-3.

### 2.13 Statistical analysis

Three repetitions were conducted in each experiment. The results were expressed as the mean ± standard deviation (SD). Differences among the various ZTs were analyzed using one-way analysis of variance (ANOVA) followed by post hoc testing, employing SPSS version 22.0 software. A p-value of less than 0.05 was considered to indicate statistical significance.

## 3 Results

### 3.1 Time of day-dependent alterations of vital signs and survival rate in septic SD rats

First, we explored the time of day-dependent effect on vital signs and survival rate in septic SD Rats. After 2 weeks acclimating of a 12 h light-dark ZT cycle (ZT0 = lights-on and ZT12 = lights-off), rats were intraperitoneal injected with LPS at ZT4, ZT10, ZT16 or ZT22 and recorded the vital signs 2h after each injection. The mortality rate was observed every 12h for 72h after LPS injection. As shown in Table 1, in LPS group, the HR levels at ZT10 LPS injection were lower than those at other three ZTs (P < 0.05). The temperature levels of ZT10 and ZT22 LPS injection were lower than other two ZTs (P < 0.05). The MAP levels of LPS group of ZT10 LPS injection were lower than those of other three ZTs (P < 0.05). As shown in Fig. 1, LPS injected at ZT10 presented the higher death rate than those at ZT 16 (P = 0.0444) and ZT22 (P = 0.0392).

**Table 1:** Time of day-dependent alterations of vital signs in septic SD Rats.

Group		HR (bpm)	T (^o^C)	MAP (mmHg)
Con	ZT4	425.6 ± 14.3	38.2 ± 0.2	63.8 ± 9.1
	ZT10	423.1 ± 13.1	38.2 ± 0.2	57.6 ± 7.0
	ZT16	464.3 ± 19.8∗#	38.3 ± 0.3	70.8 ± 4.1#
	ZT22	445.6 ± 36.9	38.2 ± 0.2	61.6 ± 8.9
LPS	ZT4	377.7 ± 16.6	37.1 ± 0.5	63.6 ± 7.3
	ZT10	323.2 ± 13.4∗	36.0 ± 0.4∗	52.3 ± 4.9∗
	ZT16	370.9 ± 18.2#	37.0 ± 0.8#	61.2 ± 7.8#
	ZT22	352.4 ± 27.3#	36.4 ± 0.7∗	70.1 ± 4.4#
	F_group_	272.0/<0.001	204.6/<0.001	2.677/0.107
	F_time_	13.23/<0.001	5.416/0.002	9.472/<0.001
	F_group*time_	5.598/0.002	4.438/0.007	3.672/0.017

Note: The difference among each ZTs was analyzed by one-way ANOVA and post hoc test.

HR = heart rate, T = temperature, MAP = mean arterial pressure.

∗ vs. ZT4, P < 0.05 within group comparison.

# vs. ZT10, P < 0.05 within group comparison.

**Fig. 1:**
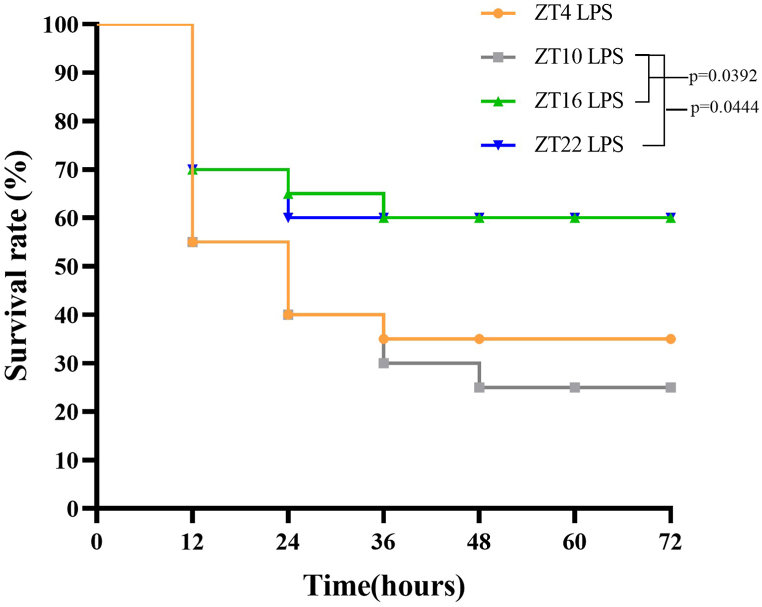
Time of day-dependent alterations of survival rate in septic SD Rats.

### 3.2 Time of day-dependent alterations of cardiac function in septic SD rats

**Fig. 2:**
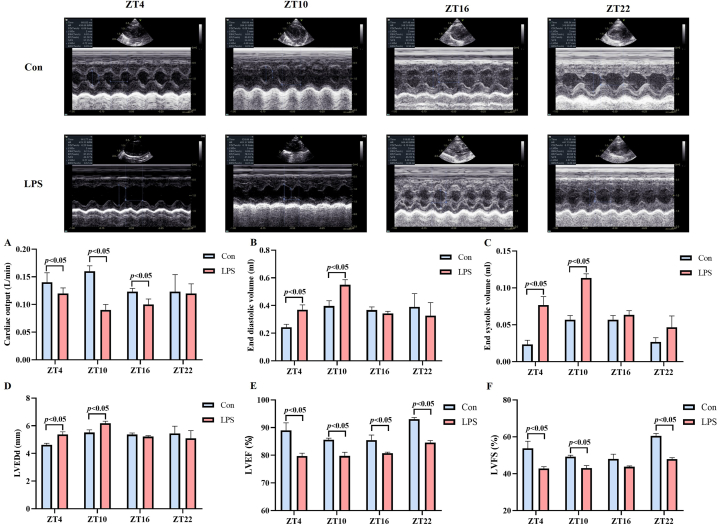
Time of day-dependent alterations of cardiac function in septic SD Rats. Echocardiography was performed 2 h after saline or LPS intraperitoneal injection. (A) Cardiac output. (B) End diastolic volume. (C) End systolic volume. (D) Left ventricular end-diastolic diameter. (E) Left ventricular ejection fraction. (F) Left ventricular fraction shortening. The error bar reflects the S.D. of at least three independent experiments.

Then, we observed that cardiac function of each group at 2h after LPS injection. As shown in Fig. 2, CO levels in the LPS group were obviously lower compared to the control group at ZT4, ZT10 and ZT16 LPS injection(P < 0.05) (Fig. 2A). The CO levels of ZT10 LPS injection were observed to be lower compared to those of ZT4 and ZT22 in the LPS group (F = 4.50, P = 0.039; post hoc test: ZT10 *vs.* ZT4 and ZT22, both P < 0.05). EDV, ESV and LVEDd levels in the LPS group were significantly higher compared to the control group of ZT4 and ZT10 injection (P < 0.05) (Fig. 2B–D). EDV, ESV and LVEDd levels in the ZT10 were higher than those of other three ZTs injection in LPS group (F_ESV_ = 22.256, P < 0.001, F_EDV_ = 10.959, P = 0.003, F_LVEDd_ = 7.952, P = 0.009; post hoc test: ZT10 *vs.* ZT4, ZT16, ZT22, all P < 0.05). LVEF levels of LPS group were significantly lower than those of control group of each ZTs injection (P < 0.05) (Fig. 2E). LVFS levels in the LPS group were significantly lower compared to the control group at ZT4, ZT10 and ZT22 injection (P < 0.05) (Fig. 2F). The LVEF and LVFS levels of ZT22 injection were higher than those at other three ZTs in LPS group (F_LVEF_ = 17.993, P = 0.001, F_LVFS_ = 19.732, P < 0.001; post hoc test: ZT10 *vs.* ZT4, ZT16, ZT22, all P < 0.05). There results indicated that the depression of cardiac function presented a time of day-dependent pattern in septic rat.

### 3.3 Time of day-dependent alterations of myocardial ferroptosis in septic SD rats

**Fig. 3:**
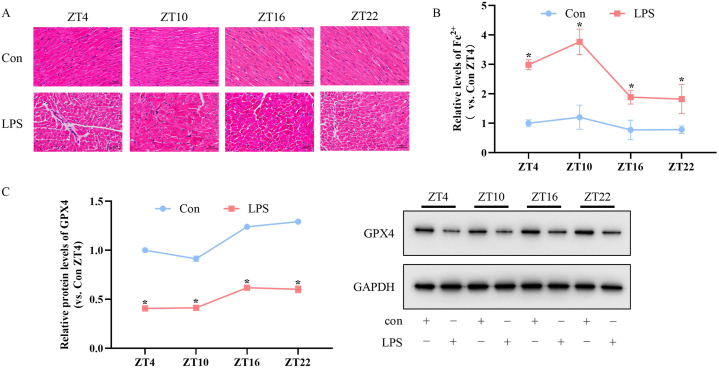
Time of day-dependent alterations of myocardial ferroptosis in septic SD Rats. (A) Representative images of inflammatory cells infiltration by HE staining (scale bar = 50 μm). (B) Contents of Fe^2+^ measured by Iron Assay Kit (DojinDo, Japan). The Fe^2+^ contents of LPS group of ZT10 injection were higher than those of other three ZTs (F = 20.23, P < 0.001; post hoc test: ZT10 *vs.* ZT4, ZT16, ZT22, all P < 0.05). (C) Protein levels of GPX4 tested by Western blot. The GPX4 protein levels of ZT4 and ZT10 LPS injection were lower than those of ZT16 and ZT22 in LPS group (F = 87.76, P < 0.001; post hoc test: ZT4 *vs.* ZT16, ZT22, both P < 0.05, and ZT10 *vs.* ZT16, ZT22, both P < 0.05). The error bar reflects the S.D. of at least three independent experiments. *P < 0.05 *vs.* Con.

Next, we evaluated the myocardial ferroptosis levels in septic rats injected with LPS at different ZTs. As shown in Fig. 3A, HE staining indicated that the infiltration of inflammatory cells in myocardial interstitium of the LPS group was markedly higher compared to the control group at each ZTs. The structural damage of LPS group of ZT10 injection was obvious than those of other three ZT points, which suggested that injection of LPS at ZT10 induced higher endotoxemia related myocardial inflammatory injury. Overload of intracellular Fe^2+^ is one of necessary conditions for ferroptosis. We found that the Fe^2+^ contents in the LPS group were obviously higher compared to the control group of each ZTs injection (P < 0.05). The Fe^2+^ contents of LPS group injected at ZT10 were higher than those of other three ZTs (F = 20.23, P < 0.001; post hoc test: ZT10 *vs.* ZT4, ZT16, ZT22, all P < 0.05) (Fig. 3B). Glutathione peroxidase 4 (GPX4), the central regulator of ferroptosisis, is an enzyme that can reduce oxidative damages, including hydrogen peroxide, organic peroxides and lipid peroxide. The results of WB showed that the GPX4 protein contents in the LPS group were markely lower compared to the control group at each ZTs. The GPX4 protein contents of ZT4 and ZT10 injection were lower than those of ZT16 and ZT22 in LPS group (F = 87.76, P < 0.001; post hoc test: ZT4 *vs.* ZT16, ZT22, both P < 0.05, and ZT10 *vs.* ZT16, ZT22, both P < 0.05) (Fig. 3C). These results showed that day-time LPS injection induced a higher level of myocardial ferroptosis.

### 3.4 Time of day-dependent alterations of Bmal-1 expression and AKT activation in heart tissue of septic SD rats

**Fig. 4:**
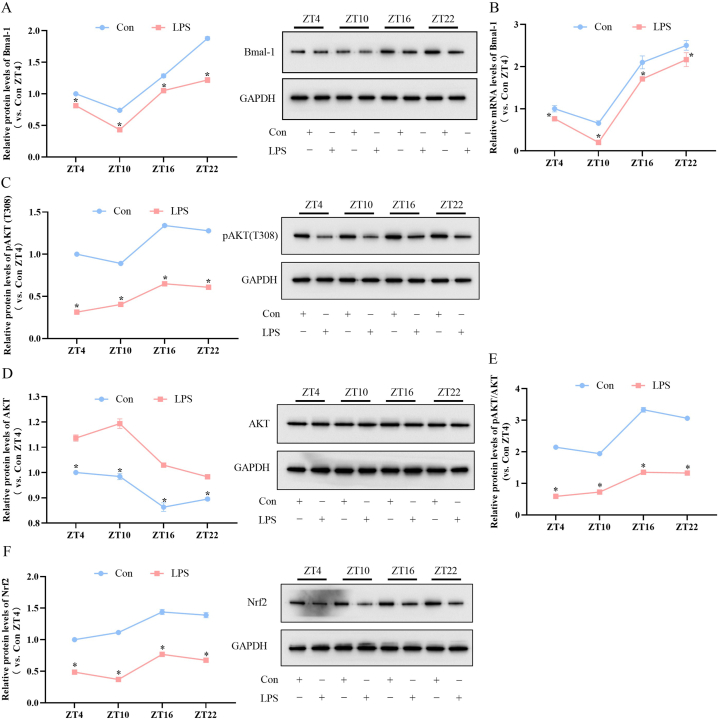
Time of day-dependent alterations of Bmal-1 expression and AKT activation in heart tissue of septic SD Rats. (A) Western blot and (B) RT-qPCR analysis showing LPS group presented low levels of Bmal-1 protein and mRNA compared with control group at each ZTs. The Bmal-1 protein levels of ZT10 injection were lower than those at other three ZTs in LPS group (F = 580.95, P < 0.001; post hoc test: ZT10 *vs.* ZT4, ZT16, ZT22, all P < 0.05). (C) pAKT and (D)AKT protein levels measured by Western blot. (E) Ratio of pAKT and AKT. The ratios of pAKT/AKT of ZT4 and ZT10 injection were lower than those of ZT16 and ZT22 in LPS group (F = 2351.94, P < 0.001; post hoc test: ZT4 *vs.* ZT16, ZT22, both P < 0.05, and ZT10 *vs.* ZT16, ZT22, both P < 0.05). (F) Nrf2 protein levels detected by Western blok. The Nrf2 protein levels of ZT10 injection was lower than those of other three ZTs in LPS group (F = 1090.77, P < 0.001; post hoc test: ZT10 *vs.* ZT4, ZT16, ZT22, all P < 0.05). The error bar reflects the S.D. of at least three independent experiments. *P < 0.05 *vs.* Con.

Next, we focused on the time of day-dependent alterations of Bmal-1 expression and AKT activation in heart tissue of septic SD Rats. First, as shown in Fig. 4A, LPS group presented low levels of Bmal-1 protein compared with control group of each ZTs injection (P < 0.05). The Bmal-1 protein levels at ZT10 were lower than those at other three ZTs in LPS group (F = 580.95, P < 0.001; post hoc test: ZT10 *vs.* ZT4, ZT16, ZT22, all P < 0.05). As shown in Fig. 4B, RT-qPCR results consisted with these changes. These results suggested that heart circadian of Bmal-1 was inhibited under endotoxemia condition. Then, we assessed the AKT activation, one of the downstream target of Bmal-1, in each group. As shown in Fig. 4C–E, pAKT/AKT ratios in the LPS group were lower compared to the control group at each ZTs. The ratios of pAKT/AKT at ZT4 and ZT10 were lower than those at ZT16 and ZT22 in LPS group (F = 2351.94, P < 0.001; post hoc test: ZT4 *vs.* ZT16, ZT22, both P < 0.05, and ZT10 *vs.* ZT16, ZT22, both P < 0.05). These suggested that the AKT activation was limited under endotoxemia condition, which might due to downregulation of Bmal-1. Next, we tested the contents of Nrf2 in each group. Nrf2 is one of the pivotal effectors in ferroptosis and regulated by AKT. As shown in Fig. 4F, the Nrf2 protein contents in the LPS group were lower compared to the control group of each ZTs injection. The Nrf2 protein levels of ZT10 injection was lower than those of other three ZTs in LPS group (F = 1090.77, P < 0.001; post hoc test: ZT10 *vs.* ZT4, ZT16, ZT22, all P < 0.05). These results mean that LPS could reduce Nrf2 expression and enhance ferroptosis. Totally, these results indicated that the circadian of Bmal-1 and its downstream AKT/Nrf2 pathway in rat heart were inhibited under endotoxemia condition.

### 3.5 Bmal-1/AKT/Nrf2 pathway mediates ferroptosis in LPS-induced H9c2 endotoxemia vitro model

**Fig. 5:**
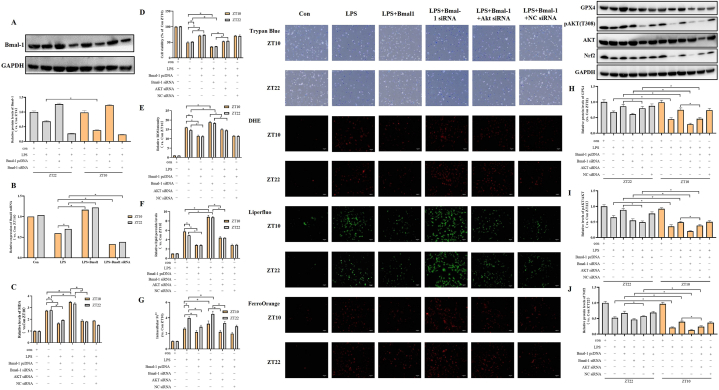
Time of day-dependent alterations of ferroptosis in LPS-induced H9c2 sepsis *vitro* model. (A) Western blot analysis showing the expressions of Bmal-1 protein in each group of ZT10 and ZT22 injection. (B) RT-qPCR showing the expressions of Bmal-1 mRNA in each group of ZT10 and ZT22 injection. (C) Levels of MDA in each group. (D) Detection of cell viability by Trypan blue staining. Dead cells were stained with blue (scale bar = 50 μm). Relative dead cell numbers in each group were expressed as the percent of Con ZT22. (E) Detection of ROS in H9c2 cells by DHE staining using fluorescence microscopy. Red means ROS staining (scale bar = 50 μm). Relative ROS levels in each group were expressed as the fold change to Con ZT22. (F) Detection of total intracellular lipid peroxide in H9c2 cells by Liperfluo staining (DojinDo, Japan) using fluorescence microscopy. Green means lipid peroxide staining (scale bar = 50 μm). Relative lipid peroxide levels in each group were expressed as the fold change to Con ZT22. (G) Detection of total intracellular Fe^2+^ in H9c2 cells by FerroOrange staining (DojinDo, Japan) using fluorescence microscopy. Red means intracellular Fe^2+^ staining (scale bar = 50 μm). Relative intracellular Fe^2+^ levels in each group were expressed as the fold change to Con ZT10 group. (H) Contents of GPX4 protein in each group of ZT10 and ZT22. (I) Ratios of pAKT/AKT in each group of ZT10 and ZT22. (J) Contents of Nrf2 protein in each group of ZT10 and ZT22 injection. The error bar reflects the S.D. of at least three independent experiments. *P < 0.05.

Then, we further validated the above results in the in *vitro* model of LPS-induced endotoxemia in H9c2 cells. For circadian clock synchronization, cells were treated by dexamethasone for 1h before experiment. Due to the results of *vivo* experiment, we selected ZT10 and ZT22 LPS administration for the *vitro* experiment. First, we tested the Bmal-1 levels after ZT10 and ZT22 LPS administration. Consistent with *vivo* experiment, we found LPS could significantly reduce the expressions of Bmal-1 protein and mRNA in H9c2 cell (Fig. 5A and B). These results confirmed that LPS-induced endotoxemia could inhibit the circadian of Bmal-1 in H9c2 cell. Next, we used trypan blue staining to evaluate the cell viability (Fig. 5D). Compared with the control group, the cell viability was lower in the LPS group (P < 0.05). In the LPS group, the cell viability at ZT10 LPS administration was lower than that at ZT22 (P < 0.05). Then, we evaluated the levels of MDA (Fig. 5C), intracellular ROS (Fig. 5E) and lipid hydroperoxides (Fig. 5F). H9c2 cells treated LPS at ZT10 LPS administration presented a higher levels of MDA, intracellular ROS and lipid hydroperoxides than those at ZT22 (P < 0.05), which mean that LPS treated at ZT10 induced a higher oxidative stress injury. Then, we measured two ferroptosis indexes, the intracellular iron (Fig. 5G) and GPX4 (Fig. 5H). LPS treated at ZT10 induced a higher levels of intracellular iron and GPX4 compared with ZT22. These results indicated that LPS-induced ferroptosis presented as the time of day-dependent alterations. Subsequently, we assessed the activation of AKT/Nrf2 (Fig. 5H and I). WB results showed that LPS treated at ZT10 LPS administration induced a lower pAKT/AKT ratios and Nrf2 contents compared with ZT22, which indicated LPS administration inhibited the AKT/Nrf2 activation as a the time of day-dependent manner. Further, we found that the up-regulation of Bmal-1 could attenuate the cell death, oxidative stress, ferroptosis and activation of AKT/Nrf2 pathway at both ZT10 and ZT22 LPS administration. Conversely, its down-regulation exhibited opposing effects. And the differences between ZT10 and ZT22 were disappeared after up-regulation or down-regulation of Bmal-1. We also found that AKT siRNA could weaken the effect of Bmal-1 pcDNA. Totally, our results suggested that LPS triggered H9c2 cell ferroptosis as a time of day-dependent manner via Bmal-1/AKT/Nrf2 pathway.

## 4 Discussion

The endogenous circadian clock regulates a multitude of physiological processes, encompassing the cycle of sleep-wake, thermoregulation, and food intake [24]. Circadian clock gene, such as BMAL-1, is also related to many dysfunctions, including immune dysfunctions and sepsis [25]. Previous studies have identified that the mortality existed daily variation in septic animals [26,27]. Mice administered with LPS during the night exhibit a lower mortality and milder inflammatory damage compared to those administered at daytime. In the present study, we built LPS-induced SICM rat model in four ZTs, including ZT4, ZT10, ZT16 and ZT22. Increase of inflammatory cells infiltration and reduction of LVEF in LPS group indicated the successful establishment of SICM model. Then, we found that LPS injection at ZT4 and ZT10 induced greater inflammatory cells infiltration in myocardial interstitium and more deteriorative cardiac function compared with injection at ZT16 and ZT22. Further, we found that the myocardial ferroptosis was more severe under LPS injection at ZT4 and ZT10 than at ZT16 and ZT22. Similar results were found in our *in-vitro* experience of LPS-induced endotoxemia in H9c2 cell. These showed that myocardial ferroptosis presented a time-dependent manner in LPS-induced endotoxemia. Ferroptosis is characterized by disruptions of iron metabolism and iron-induced lipid peroxides accumulation [25]. Ferroptosis takes part in the pathophysiological mechanisms underlying sepsis-associated organ dysfunction, such as sepsis-induced acute kidney injury [28], sepsis-induced liver injury [29], sepsis-induced acute lung injury [30], and SICM [31]. Consistent with above studies, our findings indicated that LPS administration at daytime induced a higher level of ferroptosis. The diurnal variation of ferroptosis has also been observed in other disease models, including bromobenzene-induced toxicity [32]. We believed that the circadian variations in LPS-induced ferroptosis could represent a significant advancement in the therapeutic strategies for SICM.

Substantial evidence has accumulated indicating a profound interconnection between the molecular circadian machinery and the pathophysiology of sepsis [33]. Circadian rhythms influence inflammatory processes and are, in turn, modulated by inflammation. Research has demonstrated that administering Salmonella abortus at the end of the active phase results in significantly elevated serum cortisol levels, in contrast to its administration at the onset of the active phase [34]. Our previous study indicated that LPS exacerbated ferroptosis by disrupting the circadian rhythm through Bmal1 in SICM [23]. In order to identify the mechanism of the time of day-dependent manners in LPS-induced ferroptosis, we selected the circadian clock gene, *Bmal-1*. Our results showed that Bmal-1 was low during daytime and high during the night in heart tissue of SD rat and H9c2 cell. We also found that LPS administration could inhibit expressions of Bmal-1 in rat hearts and H9c2 cells, respectively. And the daily variation of LPS-induced injury was disappeared after up-regulation or down-regulation of Bmal-1. Bmal-1 functions as a positive regulator by interacting with negative regulators such as Per1-3 and Cry1-2, thereby generating oscillations with periods approximating 24 h. It has been proved that Per2-knockout mice are under higher contents of Bmal-1 and more tolerance to LPS-induced endotoxin with lower expressions of inflammation factors [35]. The daily variation of sepsis mortality rate is lost in Bmal-1-deficent mice [36]. In hepatocytes, the deletion of Bmal-1 impedes the transcriptional response to the feeding cycle and increase the susceptibility to lethal toxic reaction induced by LPS [37]. The knock-out of myeloid cell's Bmal-1 can disrupt the circadian rhythm and decrease the survival rate in cases of LPS-induced endotoxemia [38]. In *vitro* and in *vivo* of cancer researches, it showed that Bmal-1 deletion promoted the antitumor effects of ferroptosis activators [39]. Per1, a negative regulator of Bmal-1, has been demonstrated to promote ferroptosis in the experiments of oral squamous cell carcinoma, which shows that Bmal-1 functions as a ferroptosis prohibitor [40]. In acute pancreatitis experiments, it was determined that Bmal-1 plays a protective role by mitigating inflammatory injury through the inhibition of ferroptosis-mediated HMGB1 release [41]. Thus, we harbor the idea that the Bmal-1 oscillation is the mediator in the time of day-dependent manners of LPS-triggered ferroptosis, which may act as a key point for the SICM treatment. In the realm of ICU, the circadian genes of SICM patients are influenced by prolonged exposure to artificial light, leading to heightened inflammatory responses in individuals with severe illness and critical care needs. However, the body of research examining the relationship between circadian rhythm and SICM in clinical studies remains limited.

Bmal-1 serves as a fundamental regulator of the circadian clock. Previous research has demonstrated that Bmal-1 functions as a AKT activator [42]. Our in *vivo* results suggested that the circadian of Bmal-1 and its downstream Akt/Nrf2 pathway in rat heart were inhibited under endotoxemia condition. Consistent with *in-vivo* experiment, H9c2 cell experiment demonstrated that down-regulation of AKT could weaken the protective effect of up-regulation of Bmal-1 and induce susceptibility to the toxicity of LPS. It is reported that Bmal-1 is strongly related to the dissemination of brain injury by activating AKT pathway after focal cerebral ischemia [43]. BMAL1 modulates IL-1β and the oxidative stress response in macrophages through the Nrf2 signaling pathway [44]. Our prior research demonstrated that the AKT pathway activation confers significant cardioprotective effects, notably by mitigating inflammatory damage and oxidative stress [19,21]. These myocardial effects are also implicated in SICM. Prior research has shown that the AKT pathway can enhance resistance to LPS by attenuating the expression of pro-inflammatory cytokines [45] and apoptosis [46], inhibiting the translocation of NF-kappa B nuclear [45] and autophagy [47], elevating the activity of total nitric oxide synthase [48] in sepsis. The AKT pathway functions as a suppressor of ferroptosis, with Nrf2 identified as a critical targets of AKT [49]. Activating AKT/Nrf2 pathway, phosphorylation of AKT and Nrf2 nuclear accumulation, reduced the levels of iron and MDA, and increased expressions of GSH and GPX4 in rat heart, which indicated the alleviation of ferroptosis [50]. Together, LPS administration at different ZTs encounters different levels of Bmal-1 and activation of AKT/Nrf2, which then presented different ferroptosis levels. A comprehensive examination of the circadian clock's function in ICU sepsis patients may enhance sepsis treatment strategies [51,52]. Monitoring rhythmic variables such as Bmal-1 at various points throughout the day during the initial phase of sepsis could facilitate the correlation of Bmal-1 with SICM patient outcomes, which is the concern in further clinical studies.

There are some limitation in our study. First, according to minimum quality threshold in pre-clinical sepsis studies (MQTiPSS) [53], the utilization of LPS was limited to simulating the complexities of human sepsis, as it is a conventional approach employed in experimental models of sepsis. However, our study aimed to evaluate the time-dependent effects of sepsis on circadian. Levels of Bmal-1 were measured at 2 h after sepsis model building in each ZT time. It is difficult for colon ligation puncture to satisfy the experimental requirements. Thus, we used LPS intraperitoneal injection for sepsis model. Second, we conducted measurements on only four ZTs for the purpose of circadian analysis. However, it is advisable to include a larger dataset of ZT measurements in order to effectively represent the circadian state. Third, due to financial resistance, we did not evaluate the effects of Bmal-1 overexpression or knockdown on AKT/Nrf2 signaling and ferroptosis in *vivo* model.

In summary, our findings demonstrated that ferroptosis exhibits time-of-day-dependent patterns mediated by the Bmal-1/AKT/Nrf2 pathway in both *vivo* and *vitro* models of SICM. Although further research is necessary, our results support new insights into the underlying mechanisms of SICM.

## Funding

This study was supported by the Natural Science Foundation of 10.13039/501100007956Nanjing University of Chinese Medicine (grant nos. XZR2023031). The construction project of national famous Chinese medicine experts, Prof. Zhou Min's research office (2022/07–2025/12).

## Disclosure statement

No potential conflict of interest was reported by the author(s).

## Consent for publication

Not applicable.

## Data availability statement

The datasets used and/or analyzed during the current study available from the corresponding author on reasonable request.

## CRediT authorship contribution statement

**Pei Ying-hao:** Writing – review & editing. **Yang Yu-shan:** Investigation. **Cheng Song-yi:** Investigation. **Jiang Hua:** Validation, Methodology, Investigation. **Yu Peng:** Writing – original draft, Investigation. **Chen Xiao-hu:** Writing – review & editing.

## Declaration of competing interest

The authors declare that they have no known competing financial interests or personal relationships that could have appeared to influence the work reported in this paper.
